# Dietary predictors of prenatal per- and poly-fluoroalkyl substances exposure

**DOI:** 10.1038/s41370-021-00386-6

**Published:** 2021-10-06

**Authors:** Stephanie M. Eick, Dana E. Goin, Jessica Trowbridge, Lara Cushing, Sabrina Crispo Smith, June-Soo Park, Erin DeMicco, Amy M. Padula, Tracey J. Woodruff, Rachel Morello-Frosch

**Affiliations:** 1 Program on Reproductive Health and the Environment, Department of Obstetrics, Gynecology and Reproductive Sciences, University of California, San Francisco, San Francisco, CA USA; 2 Department of Environmental Health Sciences, Fielding School of Public Health, University of California, Los Angeles, Los Angeles, CA USA; 3 Environmental Chemistry Laboratory, Department of Toxic Substances Control, California Environmental Protection Agency, Berkeley, CA USA; 4 Department of Environmental Science, Policy and Management and School of Public Health, University of California, Berkeley, Berkeley, CA USA

**Keywords:** Diet, Pregnancy, Per- and poly-fluoroalkyl substances, Nutrition

## Abstract

**Background:**

Per- and poly-fluoroalkyl substances (PFAS) are commonly detected in a variety of foods and food packaging materials. However, few studies have examined diet as a potential source of PFAS exposure during pregnancy. In the present cross-sectional study, we examined prenatal PFAS levels in relation to self-reported consumption of meats, dairy products, and processed foods during pregnancy.

**Methods:**

Participants were enrolled in the Chemicals in Our Bodies study, a demographically diverse pregnancy cohort in San Francisco, CA (*N* = 509). Diet was assessed using a self-reported interview questionnaire administered during the second trimester. Participants were asked on average how many times a day, week, or month they ate 11 different foods since becoming pregnant. Responses were categorized as at least once a week or less than once a week and foods were grouped into three categories: processed foods, dairy products, and meats. Twelve PFAS (ng/mL) were measured in second trimester serum samples. We investigated relationships between consumption of individual dairy products, meats, and processed foods and natural log-transformed PFAS using separate linear regression models adjusted for maternal age, education, race/ethnicity, and nativity.

**Results:**

Seven PFAS were detected in ≥65% of participants. Consumption of dairy milk and cheese at least once per week was moderately associated with elevated levels of perfluorononanoic acid (PFNA) and perfluorodecanoic acid (PFDeA) relative to those who ate dairy products less than once week. The strongest associations observed were with PFDeA for dairy milk (*β* = 0.2, 95% confidence interval [CI] = 0.02, 0.39) and PFNA for cheese (*β* = 0.22, 95% CI = 0.02, 0.41). Eating fish, poultry, and red meat at least once per week was associated with higher levels of perfluoroundecanoic acid, PFDeA, PFNA, and perflucorooctane sulfonic acid.

**Conclusions:**

Results indicate that consumption of animal products may contribute to elevated prenatal PFAS levels.

## Introduction

Per- and poly-fluoroalkyl substances (PFAS) are environmental chemicals that are widely used in commerce due to their oil and water repellant properties. PFAS are commonly found in non-stick cookware, food containers, and some drinking water [1], leading to ubiquitous human exposure [2]. Epidemiologic studies have shown that increasing concentrations of some PFAS, particularly perfluorooctanoic acid (PFOA) and perflucorooctane sulfonic acid (PFOS), are associated with an increased risk of developing certain cancers and pregnancy-induced hypertension [3, 4]. Of all PFAS in production in the United States (US), PFOA and PFOS have historically been produced in the largest amounts and concerns regarding adverse health effects associated with these chemicals have led to voluntary phase outs by industry in the early 2000s [5]. While PFOA and PFOS levels in the US population have subsequently declined following these phase outs, they are still widely detected, indicating that they can persist in the environment and in humans for many years [6]. In addition, newer PFAS that are structurally similar to PFOA and PFOS are being phased in and we have a limited understanding of their toxicity [7].

Given the long half-life and persistence of PFAS, identifying their exposure sources is critical to inform strategies that can reduce exposures and body burden. PFAS are magnified in the food web, and diet has been implicated as a major route for PFAS exposure [1]. In addition to humans, PFOS has been detected in polar bears in the Artic and other wildlife in remote regions [8]. Furthermore, PFAS have been detected in animals, such as fish and poultry, which are subsequently consumed by humans [9, 10], and studies have observed positive relationships between fish and poultry consumption and PFAS levels [11–14]. PFAS are also used in food packaging materials due to their oil and water-repellent properties. PFAS can latch onto foods via migration from food packaging materials, such as microwave popcorn bags and take-out pizza boxes [15, 16], and previous studies have indicated that individuals who eat foods packaged in these materials, such as pizza and salty snacks, have elevated levels of many PFAS relative to those who report never eating these foods [17]. Data from the National Health and Nutrition Examination Survey (NHANES) has also shown that consumption of fast food, pizza, and microwave popcorn is associated with higher levels of PFOA and perfluorononanoic acid (PFNA) [18].

Prior studies conducted in pregnant people have consistently shown a positive relationship between fish consumption and PFAS levels [12, 19, 20]. However, we have a limited understanding of the relationship between PFAS and other dietary predictors during pregnancy. This is particularly concerning, as many PFAS, including PFOA and PFOS, are easily transferred to the fetus via the placenta [21, 22] and have been detected in 99% of pregnant people [23]. Elevated levels of PFAS prenatally have also been associated with adverse reproductive health outcomes, such as preeclampsia, preterm birth, and reduced fetal growth [24–27]. In the present study, we examined relationships between self-reported consumption of foods and food packaging materials and serum PFAS levels in a demographically diverse cohort of pregnant people in San Francisco.

## Methods

### Study population

Participants included in this analysis were enrolled in the Chemicals in Our Bodies (CIOB) cohort. CIOB was designed to examine the cumulative effects of environmental chemical and non-chemical stressors in pregnancy and is described in detail elsewhere [26, 28]. Briefly, pregnant people were recruited during their second trimester of pregnancy at three University of California, San Francisco (UCSF) hospitals beginning in 2014. Participants recruited from Moffitt Long and Mission Bay Hospitals were economically diverse, and the majority had private health insurance. Those recruited from the Zuckerberg San Francisco General Hospital were primarily lower income and enrolled in Medi-Cal (California’s Medicaid program). Eligibility for CIOB included: ≥18 years of age, singleton pregnancy, and English or Spanish speaking. As part of the study, mothers consented to study staff accessing their medical records and all participants provided written, informed consent prior to participating. The Institutional Review Boards at UCSF (13-12160) and the University of California, Berkeley (2010-05-04) approved CIOB.

### Diet

Dietary factors were assessed using a self-reported interview questionnaire adapted from prior studies examining dietary sources of PFAS [14, 29, 30], which was administered during the second trimester (see Supplementary Material). Participants were asked how many times a day, week, or month they ate certain foods since becoming pregnant. We focused our analyses on three groups of foods and food packaging sources that have been identified as potential sources of PFAS exposure in the literature: meats, processed foods, and dairy products [1, 14, 18]. Poultry, fish, shellfish, and red meats (beef, pork, lamb, goat) were included as meats. Processed foods included take-out or delivered pizza, fast food or take-out food, food from a restaurant that is packaged in paper or cardboard, food purchased from a store that comes in paper or cardboard, French fries, microwave popcorn, and movie theater popcorn. Lastly, dairy milk, cheese, and yogurt were included as dairy products. Microwave and movie theater popcorn were combined into a single popcorn category, and fish and shellfish were combined into a single fish category. Fast food or take-out food and food from a restaurant packaged in paper or cardboard were also combined into a single take-out food category. Responses to all questions were converted to the number of times consumed per week and categorized as at least once a week or less than once a week. We categorized popcorn as less than once a month or at least once a month as very few participants reported eating popcorn at least once a week. While we examined other categories (such as never, more than never to but less than once a month, more than once a month to less than once a week, at least once a week), we focused on binary measures in our main analysis as the number of participants across groups was small.

### PFAS exposure

Twelve PFAS were measured in second trimester maternal serum samples (range 12–28 weeks’ gestation), at the Environmental Chemical Laboratory at the California Department of Toxic Substances Control (DTSC). Prior to analysis, serum samples were frozen at –80 °C. PFAS were quantified by injection onto an automated online solid phase extraction method coupled to liquid chromatography and tandem mass spectrometry. Additional details regarding the analysis of PFAS are available elsewhere [22, 28]. We restricted our analysis to PFAS detected in ≥65% of samples, which included: PFOA, PFOS, PFNA, perfluorohexanesulphonic acid (PFHxS), methyl-perfluorooctane sulfonamide acetic acid (Me-PFOSA-AcOH), perfluorodecanoic acid (PFDeA), and perfluoroundecanoic acid (PFUdA). Measurements below the method detection limit (MDL) were assigned the machine read value if one was available. If there was no machine read value, the concentration was replaced with MDL/√2. All PFAS concentrations were right skewed and natural log transformed for analysis.

### Covariates

Information regarding maternal age, education, race/ethnicity, and marital status was self-reported on the second trimester interview questionnaire. Pre-pregnancy body mass index (BMI; kg/m^2^) and parity were abstracted from the medical record. Participants whose annual household income was below the 2017 San Francisco county poverty line or who reported finding it difficult to pay for food, housing, medical care, utilities, or other basic necessities were classified as experiencing financial strain [31]. When asking about the last 12 months, participants who reported skipping meals, eating less than they should, or were hungry but did not eat because there was not enough money to buy food were classified as food insecure. Those who reported that the food they bought did not last, that there was not enough money, or if they could not afford to eat balanced meals were also considered to be food insecure [32, 33]. Information regarding nativity (US born versus foreign born) was obtained via self-report.

Covariates retained in adjusted models were chosen via a directed acyclic graph (Supplementary Fig. S1) and included race/ethnicity, maternal age, education, and nativity. These covariates have been associated with both our exposure and outcomes in our study population and in prior studies [28, 34–36]. As a sensitivity analysis, we included food insecurity as a covariate in adjusted models, as we hypothesized that it may be an additional measure of socioeconomic status (SES).

### Statistical analysis

We examined the distribution of all PFAS using descriptive statistics. Linear regression models were fit to examine unadjusted and adjusted estimates and 95% confidence intervals (CI) for the association between diet and PFAS levels. We examined QQ-plots for each model to ensure that regression residuals were normally distributed.

Socioeconomic factors may influence what types of food an individual has access to, and prior studies show that PFAS levels vary across SES [37, 38]. Therefore, we conducted an additional analysis where we examined food insecurity and financial strain as potential effect modifiers of the relationship between diet and PFAS. In these analyses, stratified models were adjusted for maternal age, race/ethnicity, education, and nativity. Given the low percentage of missing data for covariates retained in adjusted models, a complete case analysis was used for all models and analyses were conducted in R Version 4.0.1.

## Results

There were 509 participants included in our analysis. Of this group, nearly 40% were 30 years of age or older and had a graduate degree. Most participants were white (38%) or Latina (34%) and half had a normal pre-pregnancy BMI. Roughly 34% of participants experienced financial strain and 15% were food insecure (Table 1).

**Table 1:** Distribution of demographic characteristics in the Chemicals in Our Bodies Study Population (*N* = 509).

	*N* (%)
*Maternal age at enrollment (years)*	
<25	54 (11)
25–29	76 (15)
30–34	182 (36)
≥35	196 (39)
Missing	1 (0.2)
*Maternal education*	
<High school	60 (12)
High school degree or some college	138 (27)
College degree	116 (23)
Graduate degree	186 (37)
Missing	9 (1.8)
*Maternal race/ethnicity*	
White	191 (38)
Black	37 (7)
Asian/Pacific Islander	85 (17)
Latina	173 (34)
Other	14 (3)
Missing	9 (1.8)
*Nativity*	
Foreign born	213 (42)
US born	419 (52)
Missing	33 (6.5)
*Parity*	
No prior births	246 (48)
One or more prior births	254 (50)
Missing	9 (1.8)
*Marital status*	
Married	337 (66)
Living together	103 (20)
Single	59 (12)
Missing	10 (2.0)
*Pre-pregnancy body mass index (kg/m*^*2*^*)*	
Underweight (<18.5)	12 (2)
Normal (18.5–24.9)	240 (47)
Overweight (25–29.9)	129 (25)
Obese (≥30)	90 (18)
Missing	38 (7.5)
*Financial strain*	
Yes	190 (37)
No	274 (54)
Missing	45 (8.8)
*Food insecurity*	
Yes	78 (15)
No	419 (82)
Missing	12 (2.4)

Of the processed foods, take-out food was consumed the most frequently with 76.6% of participants reporting consumption of take-out food at least once a week. Take-out or delivered pizza was the least commonly consumed (11.6% at least once a week on average). Nearly all participants reported consuming dairy products at least once a week on average. Cheese was the most common (86.4%), followed by dairy milk (80.1%), and yogurt (76%). Among the meats, 85.9% and 70.1% reported eating poultry and red meat on average at least once a week, respectively (Table 2).

**Table 2:** Distribution of self-reported consumption of foods in the Chemicals in Our Bodies Study (*N* = 509).

	*N* (%)
*Processed foods*	
Pizza	
<1/week	435 (85.5)
≥1/week	59 (11.6)
Missing	15 (2.9)
Take-out food	
<1/week	101 (19.8)
≥1/week	390 (76.6)
Missing	18 (3.5)
Store bought food	
<1/week	394 (77.4)
≥1/week	96 (18.9)
Missing	19 (3.7)
French fries	
<1/week	367 (72.1)
≥1/week	124 (24.4)
Missing	18 (3.5)
Popcorn	
<1/month	313 (61.5)
≥1/month	178 (35.0)
Missing	18 (3.5)
*Dairy products*	
Dairy milk	
<1/week	80 (15.7)
≥1/week	410 (80.6)
Missing	19 (3.7)
Cheese	
<1/week	51 (10.0)
≥1/week	440 (86.4)
Missing	18 (3.5)
Yogurt	
<1/week	105 (20.6)
≥1/week	387 (76.0)
Missing	17 (3.3)
*Meats*	
Poultry	
<1/week	58 (11.4)
≥1/week	437 (85.9)
Missing	14 (2.8)
Fish and shellfish	
<1/week	169 (33.2)
≥1/week	319 (62.7)
Missing	21 (4.1)
Red meat	
<1/week	137 (26.9)
≥1/week	357 (70.1)
Missing	15 (2.9)

All seven PFAS included in our analysis had machine readable values available for >90% of samples (Table 3). Of these, PFOA and PFOS were detected at the highest concentrations, with median values of 0.8 and 2.0 ng/mL, respectively. Four PFAS were detected in <65% of samples (PFDoA, PFOSA, PFBS, Et-PFOSA-AcOH, PFHpA; Table 3).

**Table 3:** Distribution of second trimester PFAS concentrations (ng/mL) in maternal serum in the Chemicals in Our Bodies Study (*N* = 509).

	MDL	% above MDL	% machine readable	Selected percentiles		
				5th	50th	Maximum
PFNA	0.06	98.8	99.6	0.1	0.3	18.7
PFOA	0.13	99.8	100	0.3	0.8	32.2
PFHxS	0.01	100	100	0.1	0.3	4.9
PFOS	0.07	100	100	0.5	1.9	14.5
Me-PFOSA-AcOH	0.01	98.8	99.8	0.02	0.05	1.8
PFDeA	0.05	69.4	92.3	0.03	0.1	3.9
PFUdA	0.08	72.3	95.5	0.02	0.1	1.3
PFDoA	0.20	2.2	58.4	0.01	0.02	0.5
PFOSA	0.02	2.4	45.6	0.001	0.02	0.1
PFBS	0.03	0.8	54.4	0.001	0.02	0.1
Et-PFOSA-AcOH	0.01	10.6	69.7	0.001	0.01	0.1
PFHpA	0.05	12.0	68.0	0.003	0.02	0.5

To calculate percentiles, machine read values were used for observations below the MDL when available and if a machine read value was unavailable, values were replaced with the MDL/√2.

*MDL* method detection limit.

**Fig. 1:**
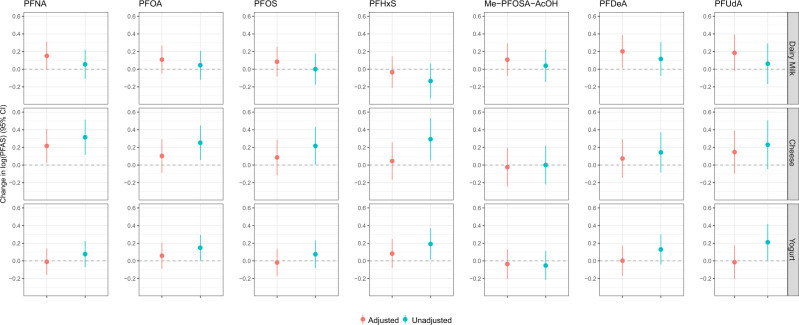
Linear regression coefficients and 95% confidence intervals indicating the mean change in natural log-transformed PFAS concentrations (ng/mL) in maternal serum in association with self-reported consumption of dairy products greater than once a week relative to less than once a week. Models adjusted for maternal age, maternal race/ethnicity, education, and nativity. CI confidence interval.

Elevated levels of PFNA, PFOA, and PFOS were observed among those who reported eating cheese at least once a week in unadjusted models (Fig. 1 and Supplementary Table S1). Only the relationship between cheese and PFNA remained relatively unchanged after adjusting for covariates (*β* = 0.23, 95% CI = 0.04, 0.42). In adjusted models, drinking dairy milk at least once a week was modestly associated with elevated levels of PFOA (*β* = 0.11, 95% CI = –0.05, 0.27), PFOS (*β* = 0.08, 95% CI = –0.08, 0.25), PFDeA (*β* = 0.20, 95% CI = 0.02, 0.39), and PFUdA (β = 0.19, 95% CI = –0.02, 0.39) (Fig. 1 and Supplementary Table S2). Eating yogurt at least once a week was associated with higher levels of PFOA, PFHxS, and PFUdA in unadjusted models only.

**Fig. 2:**
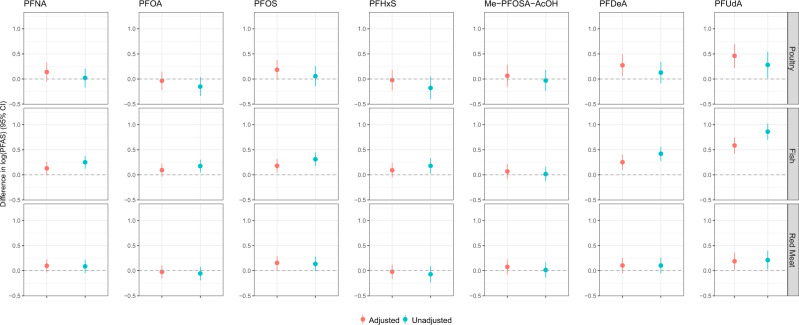
Linear regression coefficients and 95% confidence intervals indicating the mean change in natural log-transformed PFAS concentrations (ng/mL) in maternal serum in association with self-reported consumption of meats greater than once a week relative to less than once a week. Models adjusted for maternal age, maternal race/ethnicity, education, and nativity. Fish includes fish and shellfish. CI confidence interval.

Compared to those who ate fish or shellfish less than once a week, consuming fish or shellfish at least once a week was associated with elevated levels of PFNA, PFOS, PFDeA, and PFUdA in adjusted models (Fig. 2 and Supplementary Table S2). The strongest associations observed were with PFUdA (*β* = 0.58, 95% CI = 0.42, 0.74) and PFDeA (*β* = 0.25, 95% CI = 0.10, 0.40). Eating poultry and red meat at least once a week, compared to less than once a week, was also modestly associated with increasing PFOS, PFDeA, and PFUdA levels (Fig. 2).

**Fig. 3:**
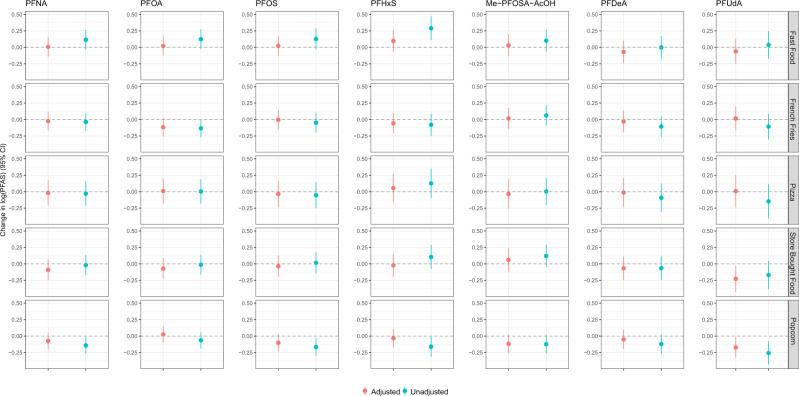
Linear regression coefficients and 95% confidence intervals indicating the mean change in natural log-transformed PFAS concentrations (ng/mL) in maternal serum in association with self-reported consumption of processed foods greater than once a week relative to less than once a week. Models adjusted for maternal age, maternal race/ethnicity, education, and nativity. Popcorn is modeled as greater than once a month relative to less than once a month. CI confidence interval.

Overall, PFAS levels were not associated with higher consumption of any of the processed foods after adjusting for covariates (Fig. 3). In unadjusted models, eating take-out food at least once a week was associated with slightly higher levels of PFOS (*β* = 0.13, 95% CI = –0.03, 0.29) and PFHxS (*β* = 0.13, 95% CI = –0.01, 0.35) relative to less than once a week. Eating microwave or movie theater popcorn at least once a month, compared to less than once a month, was moderately associated with lower levels of PFUdA (*β* = –0.26, 95% CI = –0.43, –0.08) and PFDeA (*β* = –0.12, 95% CI = –0.27, 0.02) (Fig. 3 and Supplementary Table S1). Additional adjustment for food insecurity did not noticeably change point estimates (Supplementary Table S3).

In models stratified by food insecurity, the relationships between red meat, fish consumption and PFNA, PFOS, PFDeA, and PFUdA were modestly stronger among those who were not food insecure compared to those who experienced food insecurity (Supplementary Fig. S2). Similar patterns were observed when stratifying by financial strain. In particular, PFOS, PFDeA, and PFUdA were elevated in relation to red meat and fish consumption only among those who did not experience financial strain (Supplementary Fig. S3).

## Discussion

In a large, demographically diverse pregnancy cohort in San Francisco, we observed that serum PFAS levels were positively associated with the consumption of dairy milk, cheese, fish or shellfish, red meat, and poultry after adjusting for age, race/ethnicity, education, and nativity. Furthermore, processed foods, including take-out food, French fries, and pizza, were not associated with prenatal PFAS levels in our study population. Our results suggest that animal products may be important sources of PFAS exposure during pregnancy.

Our finding that fish and shellfish consumption are associated with higher levels of long-chain PFAS, specifically PFNA, PFOS, PFDeA, and PFUdA, is supported by prior studies [11, 19, 39]. For example, in a pregnancy cohort in Spain, eating at least 5.6 servings of fish and shellfish a week was associated with elevated PFOS, PFOA, and PFNA compared to those who ate between 0 and 3.59 servings a week [40]. Similarly, in the NHANES population, consumption of fish and shellfish within the last week was positively associated with PFOA, PFNA, PFOS, and PFUdA [18, 41]. In these studies, the strongest effects observed were with PFUdA [18, 41], which is consistent with our findings and may be due to the high concentrations of PFUdA detected in fish relative to other PFAS [42]. We also observed that PFAS levels were modestly elevated among individuals who ate poultry and red meat at least once a week. This is consistent with previous studies conducted among pregnant populations in China and Spain [19, 40].

Foods that come from animals, particularly fish, have been identified as a primary source of PFAS exposure [1] and detectable levels of PFAS have been found in blood and tissue samples obtained from fish, chicken, and beef [43]. PFAS may be accumulating in fish and other animal products due to contaminated habitats, as PFAS released into the environment have been detected in drinking water for livestock, and in bays, lakes, and rivers that are home to many fish species [43, 44]. PFAS have also been detected in liver, muscle, and blood of dairy cows [45], which is consistent with our finding that levels of five PFAS were elevated among those who drank dairy milk and ate cheese at least once a week. PFAS can accumulate and bind to serum proteins in birds and fish [46] and the associations we observed with meat, seafood, and dairy products may be reflective of this. Acknowledging that there are health benefits associated with eating fish [47] and that red meat and poultry consumption is increasing globally [48], it will be important to consider seafood and white meat, as well as other animal products, as a potential source of PFAS exposure moving forward.

We observed that the relationships between fish and shellfish, red meat, and PFAS were generally stronger among individuals who did not experience food insecurity or financial strain. This is consistent with prior work in the NHANES study population showing the association between PFAS and fish and shellfish consumption was modified by household income [41]. In that study, associations were stronger among individuals with higher annual household incomes. These differences could be the result of different exposure patterns among higher SES individuals, as they may have sufficient income to purchase fish and red meat more frequently [37].

In contrast to prior findings [18], microwave popcorn and other processed foods were not a major source of PFAS exposure in our study population. Prior work shows that daily consumption of microwave popcorn is associated with elevated levels of PFAS [18], possibly due to increased contact with microwave popcorn bags, which often contain PFAS [49]. Relatively few participants in our study reported eating popcorn more than once a week, which could be why we did not observe associations between eating popcorn and PFAS levels. In our study, consumption of popcorn was analyzed as less than or equal to once a month and other studies that used similar categorizations found no association between popcorn consumption and PFAS levels. For example, in the Child Health and Development Studies, ever versus never eating microwave popcorn was not associated with PFAS levels among adult women [30]. Similarly, among children in the HOME cohort, ever versus never eating microwave popcorn in the past year was not associated with PFAS levels [11]. In contrast, the study that observed a positive association between popcorn and PFAS levels assessed diet using a 24-h recall and 12-month food frequency questionnaire, with 86% of individuals consuming popcorn within the last 12 months [18]. In addition, only 11% of participants in our study reported eating pizza on average at least once a week, and our limited sample size could be why we observed no associations between pizza consumption and PFAS levels. While not observed in our study, prior research has shown that PFAS are found in food contact paperboard (e.g., pizza boxes) [16] and that eating food from a fast food or pizza restaurant within the last 7 days is associated with elevated levels of PFOA [18]. These studies indicate that contaminated food contact materials, such as pizza boxes and microwave popcorn bags, are likely important sources of PFAS exposure on the population level. Given that PFAS are readily detected in these processed foods and packaging materials, removing PFAS from food contact materials is warranted.

An important strength of our study is that we examined numerous foods and food packaging materials in relation to PFAS exposure that are understudied, such as cheese and yogurt. In addition, our cohort included measurements of several PFAS that have not been as widely studied as PFOA and PFOS. The CIOB cohort also includes participants from diverse racial, ethnic, and socioeconomic backgrounds and our results provide important information on dietary predictors of PFAS exposure among understudied populations. We also acknowledge our limitations. Our questionnaire did not specifically ask if participants were vegetarian or vegan and prior research shows an inverse association between vegetable intake and PFAS levels [50, 51]. We attempted to estimate the number of vegetarians, but only 12 participants reported not eating any meat products. Our questionnaire was also restricted to dietary patterns during pregnancy, which may not be predictive of PFAS levels over time as PFAS bioaccumulate and diets may change more frequently. However, this exposure misclassification is likely non-differential and would bias our results toward the null. Shorter-chain PFAS were detected at low concentrations in our study and prior research has suggested that shorter-chain PFAS, such as PFBS, can bioaccumulate in the liver of fish [52]. In humans, PFAS tend to accumulate in substantial amounts across various tissues, including the lung and liver [53]. This may suggest that the serum PFAS levels in our study underestimate total bodily exposures.

Drinking water contamination is considered an important source of PFAS exposure in many communities, and people with contaminated drinking water have elevated PFAS in their blood [54, 55]. However, local San Francisco Bay Area water systems are not likely a significant source of PFAS exposure for CIOB participants; municipal water systems in the locations where most study participants live were tested for PFAS under the Environmental Protection Agency’s Unregulated Contaminant Monitoring Rule in 2016; tests of San Francisco Public Utilities Commission water sources between 2012 and 2018 did not find measurable levels of several PFAS in San Francisco or surrounding community municipal water systems [56, 57]. PFAS are also found in consumer products and building materials, representing additional exposure sources that we were unable to measure. Thus, our results may be subject to residual confounding by these factors. In addition, diet was assessed based on a self-report questionnaire asking about certain foods and foods in certain food packaging materials, which may be subject to measurement error as participants may not accurately recall frequency of all foods eaten during pregnancy. Our study had a relatively small sample size, which limited our statistical power and ability to examine certain dietary categories, including fish and shellfish, with more granularity. This imprecision is also reflected in some of the wide CIs, particularly in our analyses stratified by food insecurity and financial strain. Furthermore, we did not make any adjustments for multiple comparisons, which may increase the likelihood of chance findings. Importantly, adjusting for multiple comparisons is not always necessary in exploratory observational studies [58, 59], as it may increase the probability of type II error due to low statistical power. We did focus the interpretation of our results on identifying consistent patterns, rather than specific point estimates. Lastly, our study is cross-sectional as PFAS levels and diet were both assessed at the same second trimester visit. However, given the long half-life of PFAS, it is unlikely that our results would be subject to reverse causality.

## Conclusions

In the CIOB study population, we found that individuals who reported drinking milk and eating cheese, fish, red meat, and poultry at least once a week, compared to less than once a week, had elevated levels of PFNA, PFOS, PFDeA, and PFUdA. Processed foods, including take-out foods, pizza, and popcorn, were not associated with PFAS levels in our study. While our results contrast with prior work indicating that contact with food packaging materials may be a source of PFAS exposure, our findings may be attributed to the low level of consumption of processed foods in this population. In our study population, animal products are the main dietary source of PFAS. Given the rise of animal consumption worldwide [48], it is important to consider this source as an important dietary route of PFAS exposure during pregnancy.

## Supplementary Information

Supplementary Information
