# Supplementary material for: Dietary predictors of prenatal per- and poly-fluoroalkyl substances exposure
Source: J Expo Sci Environ Epidemiol. 2021 Oct 6;33(1):32–9. doi: 10.1038/s41370-021-00386-6 (PMC8983786; doi:10.1038/s41370-021-00386-6)
Supplement: Supplementary file 1 — Supplementary Information [file 41370_2021_386_MOESM1_ESM.docx]

Table S1. Unadjusted linear regression coefficients and 95% confidence intervals indicating the change in natural log transformed PFAS concentrations (ng/mL) in maternal serum in association with self-reported consumption of foods.

|  |  | **PFNA** | | **PFOS** | | **PFOA** | | **PFHxS** | | **Me-PFOSA-AcOH** | | **PFDeA** | | **PFUdA** | |
| --- | --- | --- | --- | --- | --- | --- | --- | --- | --- | --- | --- | --- | --- | --- | --- |
|  | **N** | **β** | **95% CI** | **β** | **95% CI** | **β** | **95% CI** | **β** | **95% CI** | **β** | **95% CI** | **β** | **95% CI** | **β** | **95% CI** |
| **Processed Foods** | | | | | | | | | | | | | | | |
| Pizza |  |  |  |  |  |  |  |  |  |  |  |  |  |  |  |
| <1/week | 435 | Ref | Ref | Ref | Ref | Ref | Ref | Ref | Ref | Ref | Ref | Ref | Ref | Ref | Ref |
| >1/week | 59 | -0.03 | (-0.22, 0.16) | -0.05 | (-0.25, 0.14) | 0 | (-0.18, 0.19) | 0.13 | (-0.1, 0.35) | 0 | (-0.2, 0.21) | -0.09 | (-0.31, 0.12) | -0.15 | (-0.41, 0.11) |
| Take-Out Food |  |  |  |  |  |  |  |  |  |  |  |  |  |  |  |
| <1/week | 101 | Ref | Ref | Ref | Ref | Ref | Ref | Ref | Ref | Ref | Ref | Ref | Ref | Ref | Ref |
| >1/week | 390 | 0.12 | (-0.03, 0.27) | 0.13 | (-0.03, 0.29) | 0.12 | (-0.02, 0.27) | 0.29 | (0.11, 0.47) | 0.1 | (-0.06, 0.27) | 0 | (-0.18, 0.17) | 0.04 | (-0.17, 0.25) |
| Store Bought Food |  |  |  |  |  |  |  |  |  |  |  |  |  |  |  |
| <1/week | 394 | Ref | Ref | Ref | Ref | Ref | Ref | Ref | Ref | Ref | Ref | Ref | Ref | Ref | Ref |
| >1/week | 96 | -0.02 | (-0.17, 0.14) | 0.02 | (-0.15, 0.18) | -0.01 | (-0.16, 0.14) | 0.1 | (-0.08, 0.29) | 0.12 | (-0.05, 0.29) | -0.07 | (-0.24, 0.11) | -0.17 | (-0.38, 0.05) |
| French Fries |  |  |  |  |  |  |  |  |  |  |  |  |  |  |  |
| <1/week | 367 | Ref | Ref | Ref | Ref | Ref | Ref | Ref | Ref | Ref | Ref | Ref | Ref | Ref | Ref |
| >1/week | 124 | -0.04 | (-0.18, 0.1) | -0.05 | (-0.2, 0.1) | -0.13 | (-0.27, 0) | -0.08 | (-0.25, 0.09) | 0.06 | (-0.09, 0.22) | -0.11 | (-0.27, 0.06) | -0.11 | (-0.3, 0.09) |
| Popcorn |  |  |  |  |  |  |  |  |  |  |  |  |  |  |  |
| <1/month | 313 | Ref | Ref | Ref | Ref | Ref | Ref | Ref | Ref | Ref | Ref | Ref | Ref | Ref | Ref |
| >1/month | 178 | -0.14 | (-0.27, -0.02) | -0.17 | (-0.3, -0.03) | -0.06 | (-0.19, 0.06) | -0.16 | (-0.31, -0.01) | -0.12 | (-0.26, 0.02) | -0.12 | (-0.27, 0.02) | -0.26 | (-0.43, -0.08) |
| **Dairy Products** | | | | | | | | | | | | | | | |
| Dairy Milk |  |  |  |  |  |  |  |  |  |  |  |  |  |  |  |
| <1/week | 80 | Ref | Ref | Ref | Ref | Ref | Ref | Ref | Ref | Ref | Ref | Ref | Ref | Ref | Ref |
| >1/week | 410 | 0.06 | (-0.11, 0.22) | 0 | (-0.17, 0.18) | 0.04 | (-0.12, 0.21) | -0.13 | (-0.33, 0.06) | 0.04 | (-0.14, 0.22) | 0.12 | (-0.08, 0.31) | 0.06 | (-0.17, 0.29) |
| Cheese |  |  |  |  |  |  |  |  |  |  |  |  |  |  |  |
| <1/week | 51 | Ref | Ref | Ref | Ref | Ref | Ref | Ref | Ref | Ref | Ref | Ref | Ref | Ref | Ref |
| >1/week | 440 | 0.31 | (0.12, 0.51) | 0.22 | (0.01, 0.43) | 0.25 | (0.06, 0.45) | 0.29 | (0.05, 0.53) | 0 | (-0.22, 0.22) | 0.14 | (-0.08, 0.37) | 0.23 | (-0.05, 0.51) |
| Yogurt |  |  |  |  |  |  |  |  |  |  |  |  |  |  |  |
| <1/week | 105 | Ref | Ref | Ref | Ref | Ref | Ref | Ref | Ref | Ref | Ref | Ref | Ref | Ref | Ref |
| >1/week | 387 | 0.08 | (-0.07, 0.23) | 0.07 | (-0.08, 0.23) | 0.15 | (0, 0.3) | 0.19 | (0.01, 0.37) | -0.05 | (-0.21, 0.11) | 0.13 | (-0.04, 0.3) | 0.21 | (0.01, 0.42) |
| **Meats** | | | | | | | | | | | | | | | |
| Poultry |  |  |  |  |  |  |  |  |  |  |  |  |  |  |  |
| <1/week | 58 | Ref | Ref | Ref | Ref | Ref | Ref | Ref | Ref | Ref | Ref | Ref | Ref | Ref | Ref |
| >1/week | 437 | 0.02 | (-0.17, 0.21) | 0.06 | (-0.14, 0.25) | -0.15 | (-0.34, 0.03) | -0.18 | (-0.4, 0.05) | -0.03 | (-0.24, 0.18) | 0.13 | (-0.09, 0.34) | 0.28 | (0.02, 0.54) |
| Fish and Shellfish |  |  |  |  |  |  |  |  |  |  |  |  |  |  |  |
| <1/week | 169 | Ref | Ref | Ref | Ref | Ref | Ref | Ref | Ref | Ref | Ref | Ref | Ref | Ref | Ref |
| >1/week | 319 | 0.25 | (0.12, 0.38) | 0.31 | (0.18, 0.44) | 0.18 | (0.05, 0.3) | 0.18 | (0.03, 0.33) | 0.01 | (-0.13, 0.16) | 0.42 | (0.28, 0.56) | 0.86 | (0.7, 1.02) |
| Red Meat |  |  |  |  |  |  |  |  |  |  |  |  |  |  |  |
| <1/week | 137 | Ref | Ref | Ref | Ref | Ref | Ref | Ref | Ref | Ref | Ref | Ref | Ref | Ref | Ref |
| >1/week | 357 | 0.09 | (-0.05, 0.22) | 0.14 | (-0.01, 0.28) | -0.06 | (-0.19, 0.08) | -0.07 | (-0.23, 0.09) | 0.01 | (-0.14, 0.16) | 0.1 | (-0.05, 0.26) | 0.21 | (0.02, 0.4) |

Abbreviations: Ref, reference.

Table S2. Adjusted linear regression coefficients and 95% confidence intervals indicating the change in natural log transformed PFAS concentrations (ng/mL) in maternal serum in association with self-reported consumption of foods.

|  |  | **PFNA** | | **PFOS** | | **PFOA** | | **PFHxS** | | **Me-PFOSA-AcOH** | | **PFDeA** | | **PFUdA** | |
| --- | --- | --- | --- | --- | --- | --- | --- | --- | --- | --- | --- | --- | --- | --- | --- |
|  | **N** | **β** | **95% CI** | **β** | **95% CI** | **β** | **95% CI** | **β** | **95% CI** | **β** | **95% CI** | **β** | **95% CI** | **β** | **95% CI** |
| **Processed Foods** | | | | | | | | | | | | | | | |
| Pizza |  |  |  |  |  |  |  |  |  |  |  |  |  |  |  |
| <1/week | 408 | Ref | Ref | Ref | Ref | Ref | Ref | Ref | Ref | Ref | Ref | Ref | Ref | Ref | Ref |
| >1/week | 51 | -0.02 | (-0.21, 0.18) | -0.03 | (-0.24, 0.17) | 0.01 | (-0.18, 0.2) | 0.05 | (-0.16, 0.27) | -0.03 | (-0.26, 0.19) | -0.01 | (-0.23, 0.21) | 0.01 | (-0.24, 0.25) |
| Take-Out Food |  |  |  |  |  |  |  |  |  |  |  |  |  |  |  |
| <1/week | 96 | Ref | Ref | Ref | Ref | Ref | Ref | Ref | Ref | Ref | Ref | Ref | Ref | Ref | Ref |
| >1/week | 359 | 0.01 | (-0.14, 0.16) | 0.02 | (-0.13, 0.18) | 0.02 | (-0.12, 0.17) | 0.1 | (-0.07, 0.26) | 0.03 | (-0.14, 0.2) | -0.07 | (-0.24, 0.1) | -0.06 | (-0.25, 0.13) |
| Store Bought Food |  |  |  |  |  |  |  |  |  |  |  |  |  |  |  |
| <1/week | 366 | Ref | Ref | Ref | Ref | Ref | Ref | Ref | Ref | Ref | Ref | Ref | Ref | Ref | Ref |
| >1/week | 88 | -0.09 | (-0.25, 0.07) | -0.03 | (-0.2, 0.13) | -0.07 | (-0.22, 0.08) | -0.02 | (-0.19, 0.15) | 0.06 | (-0.12, 0.24) | -0.07 | (-0.24, 0.11) | -0.23 | (-0.43, -0.03) |
| French Fries |  |  |  |  |  |  |  |  |  |  |  |  |  |  |  |
| <1/week | 345 | Ref | Ref | Ref | Ref | Ref | Ref | Ref | Ref | Ref | Ref | Ref | Ref | Ref | Ref |
| >1/week | 111 | -0.02 | (-0.17, 0.12) | 0 | (-0.15, 0.14) | -0.12 | (-0.26, 0.02) | -0.06 | (-0.21, 0.1) | 0.02 | (-0.15, 0.18) | -0.03 | (-0.19, 0.13) | 0.02 | (-0.16, 0.2) |
| Popcorn |  |  |  |  |  |  |  |  |  |  |  |  |  |  |  |
| <1/month | 294 | Ref | Ref | Ref | Ref | Ref | Ref | Ref | Ref | Ref | Ref | Ref | Ref | Ref | Ref |
| >1/month | 161 | -0.07 | (-0.2, 0.05) | -0.1 | (-0.23, 0.03) | 0.03 | (-0.1, 0.15) | -0.03 | (-0.17, 0.11) | -0.12 | (-0.26, 0.03) | -0.05 | (-0.19, 0.1) | -0.17 | (-0.33, -0.01) |
| **Dairy Products** | | | | | | | | | | | | | | | |
| Dairy Milk |  |  |  |  |  |  |  |  |  |  |  |  |  |  |  |
| <1/week | 73 | Ref | Ref | Ref | Ref | Ref | Ref | Ref | Ref | Ref | Ref | Ref | Ref | Ref | Ref |
| >1/week | 383 | 0.15 | (-0.01, 0.31) | 0.08 | (-0.08, 0.25) | 0.11 | (-0.05, 0.27) | -0.03 | (-0.21, 0.14) | 0.11 | (-0.08, 0.29) | 0.2 | (0.02, 0.39) | 0.19 | (-0.02, 0.39) |
| Cheese |  |  |  |  |  |  |  |  |  |  |  |  |  |  |  |
| <1/week | 50 | Ref | Ref | Ref | Ref | Ref | Ref | Ref | Ref | Ref | Ref | Ref | Ref | Ref | Ref |
| >1/week | 406 | 0.22 | (0.02, 0.41) | 0.09 | (-0.11, 0.29) | 0.1 | (-0.09, 0.29) | 0.05 | (-0.17, 0.26) | -0.02 | (-0.24, 0.2) | 0.07 | (-0.14, 0.29) | 0.15 | (-0.1, 0.39) |
| Yogurt |  |  |  |  |  |  |  |  |  |  |  |  |  |  |  |
| <1/week | 99 | Ref | Ref | Ref | Ref | Ref | Ref | Ref | Ref | Ref | Ref | Ref | Ref | Ref | Ref |
| >1/week | 358 | -0.01 | (-0.16, 0.14) | -0.02 | (-0.17, 0.14) | 0.06 | (-0.09, 0.21) | 0.08 | (-0.08, 0.25) | -0.04 | (-0.2, 0.13) | 0 | (-0.17, 0.17) | -0.02 | (-0.21, 0.18) |
| **Meats** | | | | | | | | | | | | | | | |
| Poultry |  |  |  |  |  |  |  |  |  |  |  |  |  |  |  |
| <1/week | 52 | Ref | Ref | Ref | Ref | Ref | Ref | Ref | Ref | Ref | Ref | Ref | Ref | Ref | Ref |
| >1/week | 407 | 0.14 | (-0.05, 0.33) | 0.18 | (-0.01, 0.38) | -0.04 | (-0.22, 0.15) | -0.02 | (-0.23, 0.18) | 0.06 | (-0.15, 0.28) | 0.27 | (0.06, 0.49) | 0.46 | (0.22, 0.7) |
| Fish and Shellfish |  |  |  |  |  |  |  |  |  |  |  |  |  |  |  |
| <1/week | 152 | Ref | Ref | Ref | Ref | Ref | Ref | Ref | Ref | Ref | Ref | Ref | Ref | Ref | Ref |
| >1/week | 300 | 0.13 | (0, 0.26) | 0.18 | (0.04, 0.32) | 0.09 | (-0.04, 0.22) | 0.09 | (-0.05, 0.24) | 0.07 | (-0.08, 0.22) | 0.25 | (0.1, 0.4) | 0.58 | (0.42, 0.74) |
| Red Meat |  |  |  |  |  |  |  |  |  |  |  |  |  |  |  |
| <1/week | 128 | Ref | Ref | Ref | Ref | Ref | Ref | Ref | Ref | Ref | Ref | Ref | Ref | Ref | Ref |
| >1/week | 331 | 0.1 | (-0.03, 0.23) | 0.16 | (0.02, 0.29) | -0.02 | (-0.15, 0.11) | -0.02 | (-0.17, 0.13) | 0.08 | (-0.08, 0.23) | 0.1 | (-0.05, 0.26) | 0.19 | (0.02, 0.36) |

Abbreviations: Ref, reference.

Models adjusted for maternal age, maternal education, nativity, and maternal race/ethnicity.

Table S3. Adjusted linear regression coefficients and 95% confidence intervals indicating the change in natural log transformed PFAS concentrations (ng/mL) in maternal serum in association with self-reported consumption of foods, additionally adjusted for food insecurity.

|  |  | **PFNA** | | **PFOS** | | **PFOA** | | **PFHxS** | | **Me-PFOSA-AcOH** | | **PFDeA** | | **PFUdA** | |
| --- | --- | --- | --- | --- | --- | --- | --- | --- | --- | --- | --- | --- | --- | --- | --- |
|  | **N** | **β** | **95% CI** | **β** | **95% CI** | **β** | **95% CI** | **β** | **95% CI** | **β** | **95% CI** | **β** | **95% CI** | **β** | **95% CI** |
| **Processed Foods** | | | | | | | | | | | | | | | |
| Pizza |  |  |  |  |  |  |  |  |  |  |  |  |  |  |  |
| <1/week | 408 | Ref | Ref | Ref | Ref | Ref | Ref | Ref | Ref | Ref | Ref | Ref | Ref | Ref | Ref |
| >1/week | 51 | -0.02 | (-0.21, 0.17) | -0.04 | (-0.24, 0.17) | 0.01 | (-0.18, 0.2) | 0.05 | (-0.16, 0.26) | -0.03 | (-0.25, 0.19) | -0.01 | (-0.23, 0.21) | 0.01 | (-0.24, 0.25) |
| Take-Out Food |  |  |  |  |  |  |  |  |  |  |  |  |  |  |  |
| <1/week | 96 | Ref | Ref | Ref | Ref | Ref | Ref | Ref | Ref | Ref | Ref | Ref | Ref | Ref | Ref |
| >1/week | 359 | 0 | (-0.15, 0.15) | 0.02 | (-0.14, 0.17) | 0.02 | (-0.13, 0.16) | 0.08 | (-0.08, 0.25) | 0.07 | (-0.1, 0.23) | -0.06 | (-0.24, 0.11) | -0.06 | (-0.25, 0.13) |
| Store Bought Food |  |  |  |  |  |  |  |  |  |  |  |  |  |  |  |
| <1/week | 366 | Ref | Ref | Ref | Ref | Ref | Ref | Ref | Ref | Ref | Ref | Ref | Ref | Ref | Ref |
| >1/week | 88 | -0.09 | (-0.25, 0.06) | -0.04 | (-0.2, 0.13) | -0.07 | (-0.23, 0.08) | -0.03 | (-0.2, 0.14) | 0.08 | (-0.1, 0.25) | -0.06 | (-0.24, 0.12) | -0.23 | (-0.43, -0.03) |
| French Fries |  |  |  |  |  |  |  |  |  |  |  |  |  |  |  |
| <1/week | 345 | Ref | Ref | Ref | Ref | Ref | Ref | Ref | Ref | Ref | Ref | Ref | Ref | Ref | Ref |
| >1/week | 111 | -0.02 | (-0.16, 0.12) | 0 | (-0.15, 0.14) | -0.12 | (-0.26, 0.02) | -0.06 | (-0.21, 0.1) | 0.01 | (-0.15, 0.17) | -0.03 | (-0.19, 0.13) | 0.02 | (-0.17, 0.2) |
| Popcorn |  |  |  |  |  |  |  |  |  |  |  |  |  |  |  |
| <1/month | 294 | Ref | Ref | Ref | Ref | Ref | Ref | Ref | Ref | Ref | Ref | Ref | Ref | Ref | Ref |
| >1/month | 161 | -0.07 | (-0.2, 0.05) | -0.1 | (-0.23, 0.03) | 0.03 | (-0.1, 0.15) | -0.03 | (-0.17, 0.11) | -0.12 | (-0.26, 0.02) | -0.05 | (-0.19, 0.09) | -0.17 | (-0.33, -0.01) |
| **Dairy Products** | | | | | | | | | | | | | | | |
| Dairy Milk |  |  |  |  |  |  |  |  |  |  |  |  |  |  |  |
| <1/week | 73 | Ref | Ref | Ref | Ref | Ref | Ref | Ref | Ref | Ref | Ref | Ref | Ref | Ref | Ref |
| >1/week | 383 | 0.15 | (-0.01, 0.31) | 0.08 | (-0.09, 0.25) | 0.11 | (-0.05, 0.27) | -0.04 | (-0.21, 0.14) | 0.11 | (-0.07, 0.29) | 0.2 | (0.02, 0.39) | 0.19 | (-0.02, 0.39) |
| Cheese |  |  |  |  |  |  |  |  |  |  |  |  |  |  |  |
| <1/week | 50 | Ref | Ref | Ref | Ref | Ref | Ref | Ref | Ref | Ref | Ref | Ref | Ref | Ref | Ref |
| >1/week | 406 | 0.21 | (0.02, 0.41) | 0.08 | (-0.12, 0.28) | 0.1 | (-0.09, 0.29) | 0.03 | (-0.18, 0.24) | 0.01 | (-0.21, 0.23) | 0.08 | (-0.14, 0.3) | 0.15 | (-0.1, 0.39) |
| Yogurt |  |  |  |  |  |  |  |  |  |  |  |  |  |  |  |
| <1/week | 99 | Ref | Ref | Ref | Ref | Ref | Ref | Ref | Ref | Ref | Ref | Ref | Ref | Ref | Ref |
| >1/week | 358 | -0.02 | (-0.17, 0.14) | -0.03 | (-0.18, 0.13) | 0.06 | (-0.09, 0.21) | 0.07 | (-0.1, 0.23) | -0.01 | (-0.18, 0.16) | 0.01 | (-0.16, 0.18) | -0.02 | (-0.21, 0.18) |
| **Meats** | | | | | | | | | | | | | | | |
| Poultry |  |  |  |  |  |  |  |  |  |  |  |  |  |  |  |
| <1/week | 52 | Ref | Ref | Ref | Ref | Ref | Ref | Ref | Ref | Ref | Ref | Ref | Ref | Ref | Ref |
| >1/week | 407 | 0.14 | (-0.05, 0.33) | 0.18 | (-0.01, 0.38) | -0.04 | (-0.22, 0.15) | -0.02 | (-0.22, 0.18) | 0.06 | (-0.16, 0.27) | 0.27 | (0.06, 0.49) | 0.46 | (0.22, 0.7) |
| Fish and Shellfish |  |  |  |  |  |  |  |  |  |  |  |  |  |  |  |
| <1/week | 152 | Ref | Ref | Ref | Ref | Ref | Ref | Ref | Ref | Ref | Ref | Ref | Ref | Ref | Ref |
| >1/week | 300 | 0.13 | (0, 0.26) | 0.18 | (0.04, 0.32) | 0.09 | (-0.04, 0.22) | 0.08 | (-0.06, 0.23) | 0.09 | (-0.06, 0.24) | 0.26 | (0.11, 0.41) | 0.59 | (0.43, 0.75) |
| Red Meat |  |  |  |  |  |  |  |  |  |  |  |  |  |  |  |
| <1/week | 128 | Ref | Ref | Ref | Ref | Ref | Ref | Ref | Ref | Ref | Ref | Ref | Ref | Ref | Ref |
| >1/week | 331 | 0.1 | (-0.03, 0.23) | 0.16 | (0.02, 0.29) | -0.02 | (-0.15, 0.11) | -0.02 | (-0.17, 0.12) | 0.08 | (-0.07, 0.23) | 0.11 | (-0.05, 0.26) | 0.19 | (0.02, 0.36) |

Abbreviations: Ref, reference.

Models adjusted for maternal age, maternal education, nativity, maternal race/ethnicity, and food insecurity.

Figure S1. Directed Acyclic Graph (DAG) for the association between diet and PFAS.


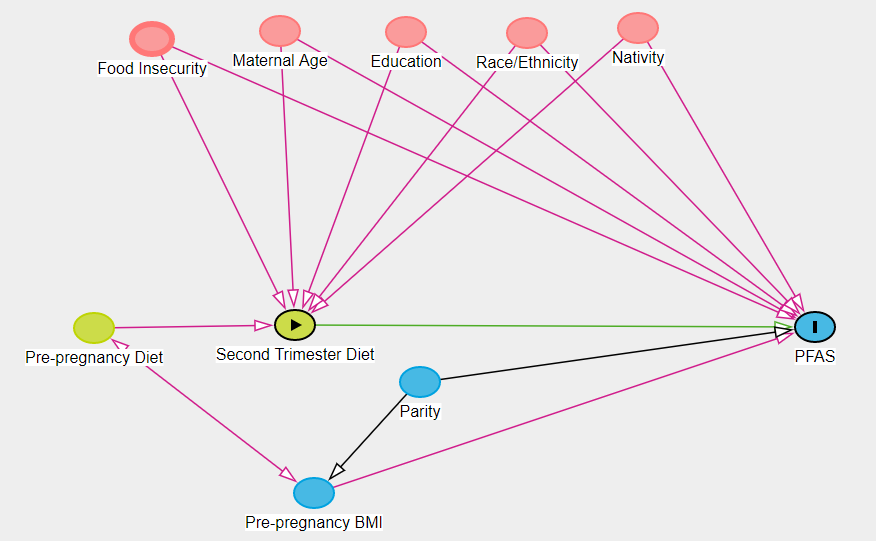


Note: Green indicates exposure, blue indicates outcome and covariates associated with outcome, red indicates covariate associated with both exposure and outcome.


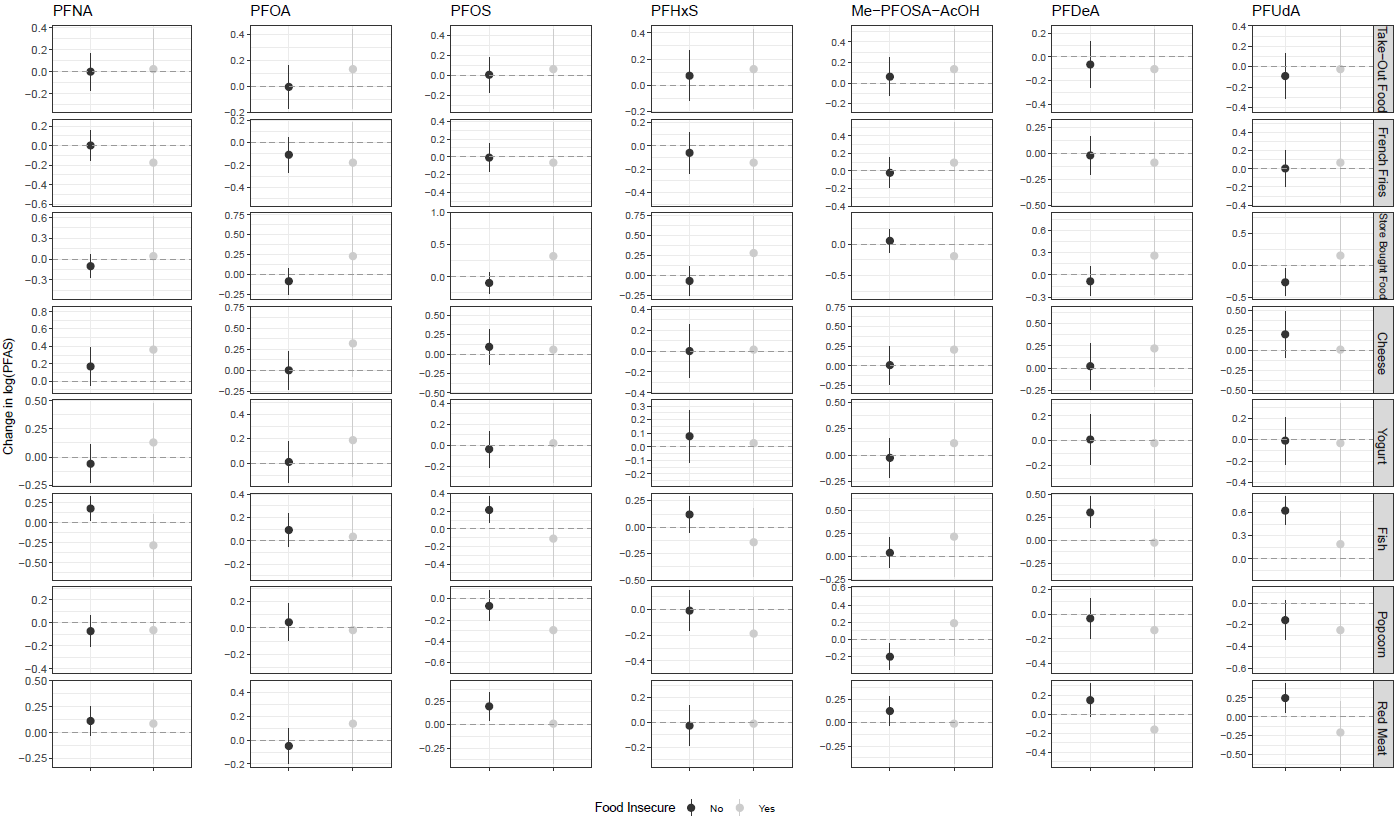


Figure S2. Adjusted linear regression coefficients and 95% confidence intervals indicating the change in natural log transformed PFAS concentrations (ng/mL) in maternal serum in association with self-reported consumption of foods stratified by food insecurity.

Note: Models adjusted for maternal age, maternal race/ethnicity, education, and nativity. Fish includes fish and shellfish.


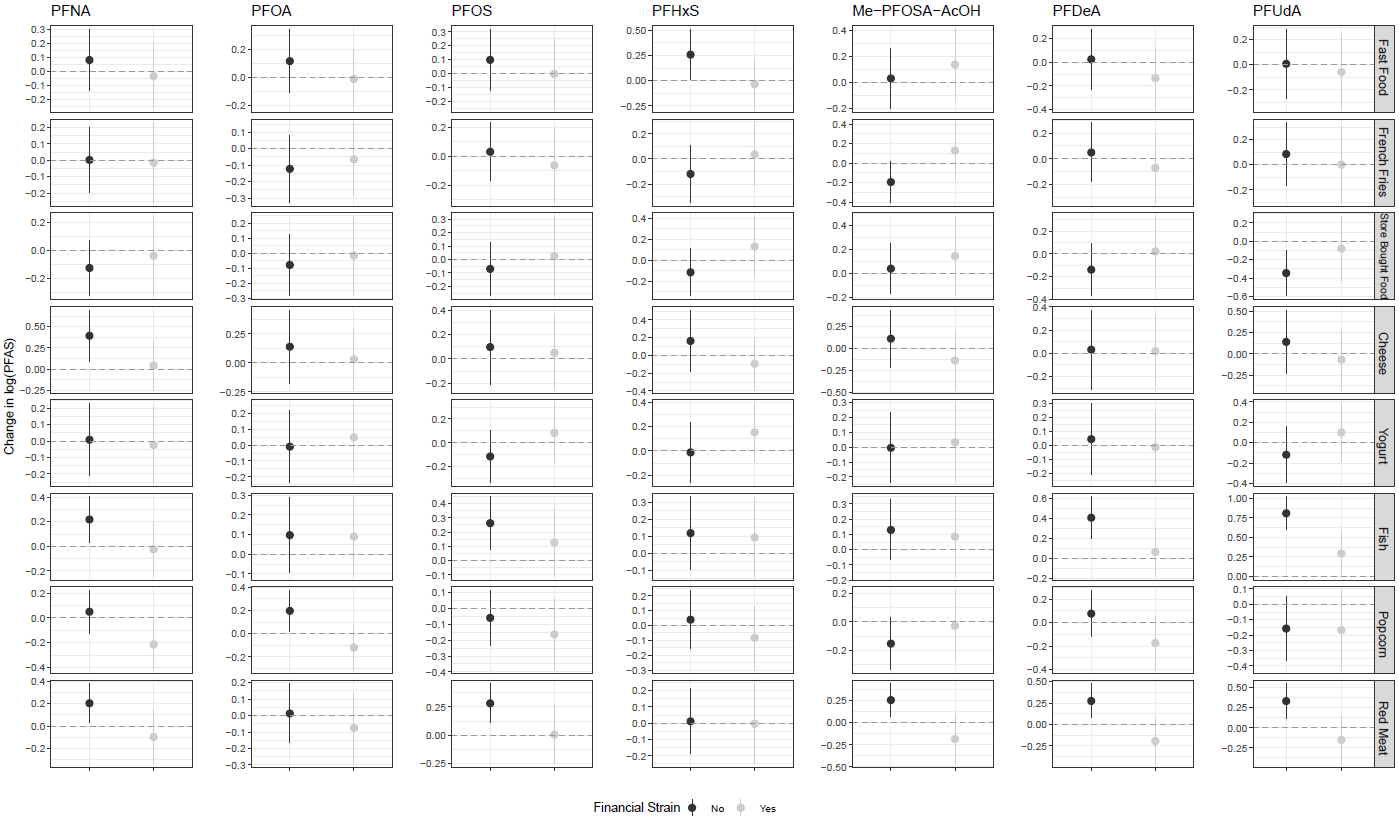


Figure S3. Adjusted linear regression coefficients and 95% confidence intervals indicating the change in natural log transformed PFAS concentrations (ng/mL) in maternal serum in association with self-reported consumption of foods stratified by financial strain.

Note: Models adjusted for maternal age, maternal race/ethnicity, education, and nativity. Fish includes fish and shellfish.
